# (*E*)-3-[(Di­methyl­amino)­methyl­idene]-4-phenyl-1*H*-1,5-benzodiazepin-2(3*H*)-one

**DOI:** 10.1107/S1600536813034739

**Published:** 2014-01-11

**Authors:** Mohamed Loughzail, Abdesselam Baouid, José A. Fernandes, Mohamed Driss, El Hassane Soumhi

**Affiliations:** a Laboratoire de Chimie Moléculaire, Département de Chimie, Faculté des Sciences-Semlalia, BP 2390, Université Cadi Ayyad, 40001, Marrakech, Morocco; b Department of Chemistry, University of Aveiro, CICECO, 3810-193, Aveiro, Portugal; c Laboratoire de Matériaux et Cristallochimie, Faculté des Sciences de Tunis, Université de Tunis ElManar, 2092 ElManar II, Tunis, Tunisia; d Equipe de Chimie des Matériaux et de l’Environnement, FSTG-Marrakech, Université Cadi Ayyad, Bd Abdelkrim Khattabi, BP. 549, Marrakech, Morocco

## Abstract

The asymmetric unit of the title compound, C_18_H_17_N_3_O, consists of two independent mol­ecules, each having an *E* conformation with respect to the C=C bond between the benzodiazepinone and di­methyl­amine groups. In the crystal, the two independent mol­ecules are linked into a dimer by a pair of N—H⋯O hydrogen bonds.

## Related literature   

For background to natural benzodiazepines and their properties, see: Di Braccio *et al.* (2001 ▶); Kavita *et al.* (1988 ▶). For the synthesis, see: Nardi *et al.* (1973 ▶). For a related structure, see: Loughzail *et al.* (2014 ▶).
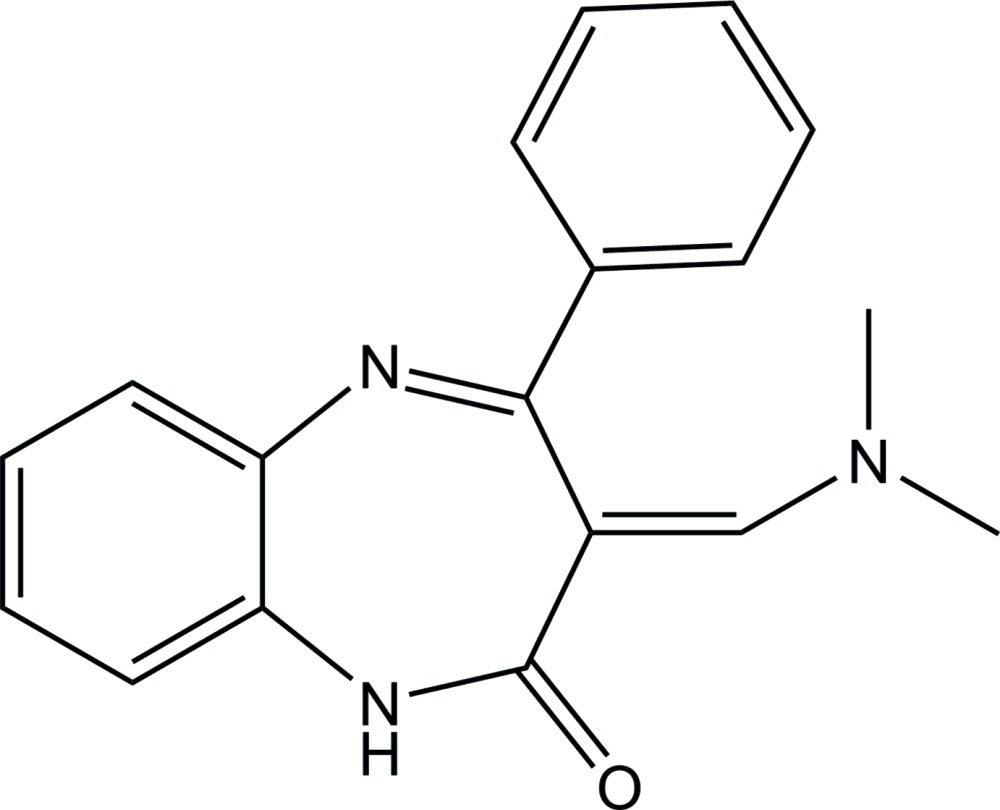


## Experimental   

### 

#### Crystal data  

C_18_H_17_N_3_O
*M* *_r_* = 291.35
Monoclinic, 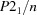

*a* = 11.281 (2) Å
*b* = 14.005 (4) Å
*c* = 20.124 (3) Å
β = 95.97 (1)°
*V* = 3162.1 (12) Å^3^
*Z* = 8
Mo *K*α radiation
μ = 0.08 mm^−1^
*T* = 300 K
0.3 × 0.15 × 0.1 mm

#### Data collection  

Enraf–Nonius CAD-4 diffractometer
Absorption correction: ψ scan (North *et al.*, 1968 ▶) *T*_min_ = 0.521, *T*_max_ = 0.992
8620 measured reflections
6879 independent reflections
3575 reflections with *I* > 2σ(*I*)
*R*_int_ = 0.034
2 standard reflections every 60 min intensity decay: 1%

#### Refinement  

*R*[*F* ^2^ > 2σ(*F* ^2^)] = 0.052
*wR*(*F* ^2^) = 0.158
*S* = 1.01
6879 reflections
402 parameters
H-atom parameters constrained
Δρ_max_ = 0.29 e Å^−3^
Δρ_min_ = −0.23 e Å^−3^

### 

Data collection: *CAD-4 EXPRESS* (Enraf–Nonius, 1989 ▶); cell refinement: *CAD-4 EXPRESS*; data reduction: *MolEN* (Fair, 1990 ▶); program(s) used to solve structure: *SHELXS97* (Sheldrick, 2008 ▶); program(s) used to refine structure: *SHELXL97* (Sheldrick, 2008 ▶); molecular graphics: *ORTEP-3 for Windows* (Farrugia, 2012 ▶); software used to prepare material for publication: *WinGX* (Farrugia, 2012 ▶).

## Supplementary Material

Crystal structure: contains datablock(s) I, hassi4. DOI: 10.1107/S1600536813034739/is5330sup1.cif

Structure factors: contains datablock(s) I. DOI: 10.1107/S1600536813034739/is5330Isup2.hkl

Supporting information file. DOI: 10.1107/S1600536813034739/is5330Isup3.cml

CCDC reference:

Additional supporting information:  crystallographic information; 3D view; checkCIF report

## Figures and Tables

**Table 1:** Hydrogen-bond geometry (Å, °)

*D*—H⋯*A*	*D*—H	H⋯*A*	*D*⋯*A*	*D*—H⋯*A*
N1—H*N*1⋯O2^i^	0.86	2.17	2.861 (2)	137
N4—H*N*4⋯O1^ii^	0.86	2.41	2.939 (2)	121

Symmetry codes: (i) 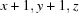; (ii) 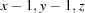.

**Fig. 1.**
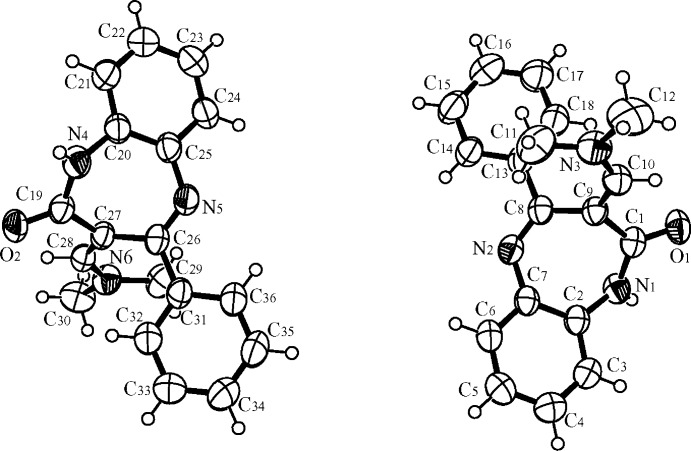
The molecule structure of the title compound with 50% probability ellipsoids.

## supplementary crystallographic information

### 1. Comment

The benzodiazepine nucleus is a pharmacophoric scaffold and represents a class of heterocycles with a wide range of applications. In particular, several representative benzodiazepines possess significant biological activity as antiviral (Di Braccio *et al.*, 2001), antipsychotic agents (Kavita *et al.*, 1988) and may be considered support for the synthesis of more active heterocyclic systems. Research in this area is highly active being directed towards the synthesis of compounds with enhanced pharmacological activity. Following the research efforts from some of us concerning novel synthetic pathways of new benzodiazepines (Loughzail *et al.*, 2014).

The reaction of *N*,*N*-dimethylformamide dimethylacetal (DMF-DMA) in 4-phenyl-1*H*-1,5-benzodiazepin-2(3*H*)-one (Nardi *et al.*, 1973) at reflux leads to the formation of the title compound, (*E*)-3-[(dimethylamino)methylene]-4-phenyl-1*H*-1,5-benzodiazepin-2(3*H*)-one with a good yield of 75%. The main geometric feature determined by X-ray diffraction is in good agreement with that observed in a similar compound (Loughzail *et al.*, 2014).

### 2. Experimental

A mixture of 0.47 g (1.98 mmol) of 4-phenyl-1*H*-1,5-benzodiazepin-2(3*H*)-one in 4.5 ml of dimethylformamide-dimethylacetal (DMF-DMA) was stirred at 100 °C for 4 h and then cooled to ambient temperature. Filtration and washing with a little cold diethyl ether gave 0.46 g of (*E*)-3-[(dimethylamino)methylene]-4-phenyl-1*H*-1,5-benzodiazepin-2(3*H*)-one. The product obtained was recrystallized from diethyl ether. Yield: 75%. Melting point: 497–498 K. ^1^H NMR (CDCl_3_, 300 MHz): δ 2.55 (s, 6H, (CH_3_)_2_N), 2.80 (s, 1H, NH), 6.32 (s, 1H, C=CH—N), 7.04–7.92 (m, 9H, H—Ar); ^13^C NMR (CDCl_3_, 75 MHz): δ 43.50 (N(CH_3_)_2_), 98.02 (C=), 121.47–141.86 (12 C-Ar), 151.18 (CH—N(CH_3_)_2_), 167.40 (Ph—C=N), 178.57 (CO). MS (m/*z*, %): 292 ([*M*+H]^+^.

### 3. Refinement

All H-atoms were located in a difference map and then refined using a riding model with N—H = 0.86 Å and C—H = 0.93–0.96 Å, and with *U*_iso_(H) = 1.2*U*_eq_(N, C) or 1.5*U*_eq_(C_methyl_).

### Crystal data

**Table:**

C_18_H_17_N_3_O	*F*(000) = 1232
*M**_r_* = 291.35	*D*_x_ = 1.224 Mg m^−^^3^
Monoclinic, *P*2_1_/*n*	Mo *K*α radiation, λ = 0.71073 Å
Hall symbol: -P 2yn	Cell parameters from 25 reflections
*a* = 11.281 (2) Å	θ = 10–15°
*b* = 14.005 (4) Å	µ = 0.08 mm^−^^1^
*c* = 20.124 (3) Å	*T* = 300 K
β = 95.97 (1)°	Prism, colourless
*V* = 3162.1 (12) Å^3^	0.3 × 0.15 × 0.1 mm
*Z* = 8	

### Data collection

**Table:**

Enraf–Nonius CAD-4 diffractometer	3575 reflections with *I* > 2σ(*I*)
Radiation source: fine-focus sealed tube	*R*_int_ = 0.034
Graphite monochromator	θ_max_ = 27.0°, θ_min_ = 2.0°
ω/2θ scans	*h* = −14→2
Absorption correction: ψ scan (North *et al.*, 1968)	*k* = −1→17
*T*_min_ = 0.521, *T*_max_ = 0.992	*l* = −25→25
8620 measured reflections	2 standard reflections every 60 min
6879 independent reflections	intensity decay: 1%

### Refinement

**Table:**

Refinement on *F*^2^	Secondary atom site location: difference Fourier map
Least-squares matrix: full	Hydrogen site location: difference Fourier map
*R*[*F*^2^ > 2σ(*F*^2^)] = 0.052	H-atom parameters constrained
*wR*(*F*^2^) = 0.158	*w* = 1/[σ^2^(*F*_o_^2^) + (0.0688*P*)^2^ + 0.4349*P*] where *P* = (*F*_o_^2^ + 2*F*_c_^2^)/3
*S* = 1.01	(Δ/σ)_max_ < 0.001
6879 reflections	Δρ_max_ = 0.29 e Å^−^^3^
402 parameters	Δρ_min_ = −0.23 e Å^−^^3^
0 restraints	Extinction correction: *SHELXL97* (Sheldrick, 2008), Fc^*^=kFc[1+0.001xFc^2^λ^3^/sin(2θ)]^-1/4^
Primary atom site location: structure-invariant direct methods	Extinction coefficient: 0.016 (2)

### Special details

**Table:**

Geometry. All e.s.d.'s (except the e.s.d. in the dihedral angle between two l.s. planes) are estimated using the full covariance matrix. The cell e.s.d.'s are taken into account individually in the estimation of e.s.d.'s in distances, angles and torsion angles; correlations between e.s.d.'s in cell parameters are only used when they are defined by crystal symmetry. An approximate (isotropic) treatment of cell e.s.d.'s is used for estimating e.s.d.'s involving l.s. planes.
Refinement. Refinement of *F*^2^ against ALL reflections. The weighted *R*-factor *wR* and goodness of fit *S* are based on *F*^2^, conventional *R*-factors *R* are based on *F*, with *F* set to zero for negative *F*^2^. The threshold expression of *F*^2^ > σ(*F*^2^) is used only for calculating *R*-factors(gt) *etc*. and is not relevant to the choice of reflections for refinement. *R*-factors based on *F*^2^ are statistically about twice as large as those based on *F*, and *R*- factors based on ALL data will be even larger.

### Fractional atomic coordinates and isotropic or equivalent isotropic displacement parameters (Å^2^)

**Table:**

	*x*	*y*	*z*	*U*_iso_*/*U*_eq_	
O1	0.87561 (14)	0.89599 (11)	0.18529 (8)	0.0622 (4)	
O2	0.10910 (14)	−0.10731 (12)	0.31490 (8)	0.0642 (4)	
N1	0.90374 (15)	0.78432 (12)	0.26535 (9)	0.0539 (5)	
HN1	0.9796	0.7923	0.2677	0.065*	
N2	0.74209 (15)	0.62029 (12)	0.23855 (9)	0.0511 (4)	
N3	0.51730 (17)	0.83342 (15)	0.14714 (10)	0.0680 (6)	
N4	0.08974 (16)	0.00488 (14)	0.23437 (10)	0.0601 (5)	
HN4	0.0145	0.0109	0.2371	0.072*	
N5	0.28648 (17)	0.14261 (13)	0.24917 (10)	0.0567 (5)	
N6	0.47471 (16)	−0.10080 (14)	0.32360 (10)	0.0585 (5)	
C1	0.83674 (18)	0.82566 (15)	0.21290 (11)	0.0486 (5)	
C2	0.85993 (18)	0.72976 (14)	0.31600 (11)	0.0486 (5)	
C3	0.9039 (2)	0.75029 (17)	0.38144 (12)	0.0652 (6)	
H3	0.9601	0.7986	0.3898	0.078*	
C4	0.8659 (3)	0.7007 (2)	0.43365 (13)	0.0836 (9)	
H4	0.8957	0.7155	0.4773	0.100*	
C5	0.7829 (3)	0.6283 (2)	0.42131 (14)	0.0809 (8)	
H5	0.7547	0.5957	0.4567	0.097*	
C6	0.7425 (2)	0.60485 (16)	0.35695 (12)	0.0623 (6)	
H6	0.6893	0.5544	0.3492	0.075*	
C7	0.77890 (18)	0.65458 (14)	0.30303 (11)	0.0477 (5)	
C8	0.71591 (17)	0.67685 (15)	0.18894 (11)	0.0469 (5)	
C9	0.71974 (18)	0.78242 (14)	0.19149 (10)	0.0477 (5)	
C10	0.6341 (2)	0.84542 (16)	0.16682 (11)	0.0549 (6)	
H10	0.6611	0.9077	0.1629	0.066*	
C11	0.4533 (2)	0.7461 (2)	0.15408 (15)	0.0837 (8)	
H111	0.4967	0.7066	0.1872	0.126*	
H211	0.4443	0.7131	0.1121	0.126*	
H311	0.3761	0.7602	0.1676	0.126*	
C12	0.4456 (3)	0.9158 (2)	0.12312 (16)	0.0989 (10)	
H112	0.4955	0.9713	0.1234	0.148*	
H212	0.3840	0.9264	0.1518	0.148*	
H312	0.4102	0.9038	0.0784	0.148*	
C13	0.68919 (18)	0.63175 (15)	0.12197 (11)	0.0512 (5)	
C14	0.6549 (2)	0.53721 (17)	0.11563 (13)	0.0635 (6)	
H14	0.6437	0.5018	0.1536	0.076*	
C15	0.6372 (2)	0.4951 (2)	0.05380 (15)	0.0791 (8)	
H15	0.6151	0.4312	0.0503	0.095*	
C16	0.6517 (2)	0.5463 (2)	−0.00301 (14)	0.0776 (8)	
H16	0.6382	0.5174	−0.0447	0.093*	
C17	0.6853 (3)	0.6380 (2)	0.00206 (14)	0.0797 (8)	
H17	0.6958	0.6727	−0.0363	0.096*	
C18	0.7045 (2)	0.68149 (18)	0.06404 (12)	0.0703 (7)	
H18	0.7281	0.7451	0.0668	0.084*	
C19	0.15585 (18)	−0.04443 (15)	0.28393 (11)	0.0500 (5)	
C20	0.13736 (19)	0.04637 (15)	0.17907 (11)	0.0531 (5)	
C21	0.0847 (2)	0.02598 (18)	0.11569 (13)	0.0697 (7)	
H21	0.0178	−0.0131	0.1106	0.084*	
C22	0.1294 (3)	0.0624 (2)	0.06001 (14)	0.0822 (8)	
H22	0.0942	0.0465	0.0176	0.099*	
C23	0.2269 (3)	0.1228 (2)	0.06716 (14)	0.0815 (8)	
H23	0.2584	0.1467	0.0296	0.098*	
C24	0.2770 (2)	0.14734 (18)	0.13008 (13)	0.0701 (7)	
H24	0.3405	0.1900	0.1347	0.084*	
C25	0.2344 (2)	0.10956 (15)	0.18699 (12)	0.0539 (6)	
C26	0.31176 (18)	0.08457 (15)	0.29803 (11)	0.0497 (5)	
C27	0.28278 (18)	−0.01887 (14)	0.29681 (10)	0.0467 (5)	
C28	0.35540 (19)	−0.09421 (15)	0.31387 (10)	0.0499 (5)	
H28	0.3156	−0.1512	0.3199	0.060*	
C29	0.5536 (2)	−0.0227 (2)	0.31182 (14)	0.0718 (7)	
H129	0.5744	0.0112	0.3528	0.108*	
H229	0.5142	0.0199	0.2793	0.108*	
H329	0.6245	−0.0474	0.2955	0.108*	
C30	0.5312 (2)	−0.19260 (19)	0.33861 (13)	0.0728 (7)	
H130	0.5894	−0.1863	0.3767	0.109*	
H230	0.5695	−0.2138	0.3008	0.109*	
H330	0.4719	−0.2384	0.3482	0.109*	
C31	0.3668 (2)	0.12737 (16)	0.36159 (11)	0.0536 (6)	
C32	0.3746 (2)	0.07799 (18)	0.42135 (12)	0.0730 (7)	
H32	0.3446	0.0162	0.4223	0.088*	
C33	0.4262 (3)	0.1191 (2)	0.47966 (14)	0.0976 (11)	
H33	0.4293	0.0854	0.5196	0.117*	
C34	0.4727 (3)	0.2090 (2)	0.47906 (15)	0.1087 (12)	
H34	0.5081	0.2364	0.5183	0.130*	
C35	0.4668 (3)	0.2583 (2)	0.42020 (15)	0.1003 (11)	
H35	0.4987	0.3194	0.4196	0.120*	
C36	0.4145 (3)	0.21896 (18)	0.36220 (13)	0.0740 (7)	
H36	0.4107	0.2539	0.3227	0.089*	

### Atomic displacement parameters (Å^2^)

**Table:**

	*U*^11^	*U*^22^	*U*^33^	*U*^12^	*U*^13^	*U*^23^
O1	0.0509 (9)	0.0479 (8)	0.0874 (11)	−0.0082 (7)	0.0046 (8)	0.0143 (8)
O2	0.0491 (9)	0.0686 (10)	0.0742 (11)	−0.0202 (8)	0.0027 (8)	0.0177 (9)
N1	0.0366 (9)	0.0559 (11)	0.0683 (12)	−0.0045 (8)	0.0019 (8)	0.0091 (9)
N2	0.0454 (10)	0.0447 (10)	0.0626 (12)	−0.0035 (8)	0.0027 (8)	−0.0017 (9)
N3	0.0468 (12)	0.0712 (13)	0.0834 (15)	0.0108 (10)	−0.0054 (10)	0.0044 (11)
N4	0.0365 (9)	0.0664 (12)	0.0765 (13)	−0.0051 (9)	0.0013 (9)	0.0148 (10)
N5	0.0551 (11)	0.0481 (10)	0.0658 (12)	−0.0115 (9)	0.0012 (9)	0.0047 (9)
N6	0.0420 (10)	0.0619 (12)	0.0709 (12)	0.0019 (9)	0.0032 (9)	0.0023 (10)
C1	0.0413 (11)	0.0409 (11)	0.0644 (14)	0.0021 (10)	0.0093 (10)	−0.0022 (10)
C2	0.0438 (11)	0.0425 (11)	0.0600 (13)	0.0008 (10)	0.0077 (10)	0.0005 (10)
C3	0.0710 (16)	0.0593 (14)	0.0645 (16)	−0.0153 (13)	0.0033 (12)	−0.0046 (12)
C4	0.110 (2)	0.0819 (19)	0.0580 (16)	−0.0237 (18)	0.0056 (15)	−0.0044 (14)
C5	0.106 (2)	0.0754 (18)	0.0628 (17)	−0.0220 (17)	0.0155 (15)	0.0092 (14)
C6	0.0689 (16)	0.0502 (13)	0.0682 (16)	−0.0122 (12)	0.0097 (12)	0.0038 (12)
C7	0.0445 (12)	0.0385 (11)	0.0603 (14)	0.0037 (9)	0.0061 (10)	0.0010 (10)
C8	0.0345 (10)	0.0446 (11)	0.0613 (13)	−0.0015 (9)	0.0042 (9)	0.0022 (10)
C9	0.0408 (11)	0.0446 (11)	0.0576 (13)	−0.0010 (10)	0.0041 (9)	0.0019 (10)
C10	0.0491 (13)	0.0514 (13)	0.0639 (14)	0.0027 (10)	0.0044 (11)	0.0032 (11)
C11	0.0438 (14)	0.102 (2)	0.104 (2)	−0.0068 (15)	0.0016 (13)	−0.0067 (17)
C12	0.0777 (19)	0.103 (2)	0.110 (2)	0.0393 (18)	−0.0192 (17)	0.0020 (19)
C13	0.0387 (11)	0.0495 (12)	0.0645 (14)	−0.0028 (10)	0.0011 (10)	−0.0013 (11)
C14	0.0614 (15)	0.0550 (14)	0.0714 (16)	−0.0099 (12)	−0.0057 (12)	−0.0020 (12)
C15	0.082 (2)	0.0627 (16)	0.089 (2)	−0.0123 (15)	−0.0093 (16)	−0.0156 (15)
C16	0.0734 (18)	0.087 (2)	0.0711 (18)	−0.0075 (16)	0.0034 (14)	−0.0215 (16)
C17	0.092 (2)	0.0838 (19)	0.0646 (17)	−0.0135 (17)	0.0120 (15)	−0.0045 (15)
C18	0.0849 (19)	0.0615 (15)	0.0653 (16)	−0.0156 (14)	0.0109 (13)	−0.0039 (13)
C19	0.0417 (11)	0.0479 (12)	0.0600 (13)	−0.0059 (10)	0.0034 (10)	0.0004 (11)
C20	0.0448 (12)	0.0485 (12)	0.0648 (14)	−0.0028 (10)	0.0006 (10)	0.0104 (11)
C21	0.0643 (15)	0.0639 (15)	0.0769 (18)	−0.0145 (13)	−0.0126 (13)	0.0119 (13)
C22	0.092 (2)	0.0830 (19)	0.0667 (17)	−0.0213 (17)	−0.0142 (15)	0.0137 (15)
C23	0.091 (2)	0.085 (2)	0.0670 (17)	−0.0198 (17)	0.0016 (15)	0.0234 (15)
C24	0.0685 (16)	0.0655 (16)	0.0743 (17)	−0.0169 (13)	−0.0026 (13)	0.0227 (13)
C25	0.0505 (13)	0.0431 (11)	0.0670 (15)	−0.0024 (10)	0.0001 (11)	0.0091 (11)
C26	0.0404 (11)	0.0474 (12)	0.0619 (14)	−0.0084 (10)	0.0082 (10)	0.0004 (11)
C27	0.0399 (11)	0.0450 (11)	0.0546 (12)	−0.0071 (9)	0.0023 (9)	0.0016 (9)
C28	0.0465 (12)	0.0484 (12)	0.0544 (13)	−0.0078 (10)	0.0034 (10)	0.0024 (10)
C29	0.0438 (13)	0.0841 (18)	0.0881 (18)	−0.0093 (13)	0.0100 (12)	−0.0039 (15)
C30	0.0660 (16)	0.0797 (18)	0.0723 (16)	0.0218 (14)	0.0058 (13)	0.0049 (14)
C31	0.0510 (13)	0.0514 (13)	0.0596 (14)	−0.0110 (10)	0.0118 (10)	−0.0028 (11)
C32	0.092 (2)	0.0630 (16)	0.0647 (16)	−0.0243 (15)	0.0117 (14)	0.0000 (13)
C33	0.142 (3)	0.094 (2)	0.0561 (16)	−0.041 (2)	0.0066 (17)	−0.0020 (15)
C34	0.158 (3)	0.103 (2)	0.0644 (18)	−0.064 (2)	0.0082 (19)	−0.0160 (17)
C35	0.144 (3)	0.0776 (19)	0.080 (2)	−0.058 (2)	0.013 (2)	−0.0136 (16)
C36	0.0922 (19)	0.0621 (15)	0.0684 (16)	−0.0279 (15)	0.0123 (14)	−0.0016 (13)

### Geometric parameters (Å, º)

**Table:**

O1—C1	1.234 (2)	C14—C15	1.372 (3)
O2—C19	1.229 (2)	C14—H14	0.9300
N1—C1	1.362 (3)	C15—C16	1.373 (4)
N1—C2	1.404 (3)	C15—H15	0.9300
N1—HN1	0.8600	C16—C17	1.341 (4)
N2—C8	1.285 (3)	C16—H16	0.9300
N2—C7	1.405 (3)	C17—C18	1.385 (3)
N3—C10	1.346 (3)	C17—H17	0.9300
N3—C11	1.434 (3)	C18—H18	0.9300
N3—C12	1.462 (3)	C19—C27	1.473 (3)
N4—C19	1.369 (3)	C20—C21	1.380 (3)
N4—C20	1.410 (3)	C20—C25	1.404 (3)
N4—HN4	0.8600	C21—C22	1.374 (4)
N5—C26	1.285 (3)	C21—H21	0.9300
N5—C25	1.404 (3)	C22—C23	1.383 (4)
N6—C28	1.343 (3)	C22—H22	0.9300
N6—C29	1.445 (3)	C23—C24	1.375 (3)
N6—C30	1.452 (3)	C23—H23	0.9300
C1—C9	1.475 (3)	C24—C25	1.392 (3)
C2—C3	1.388 (3)	C24—H24	0.9300
C2—C7	1.401 (3)	C26—C27	1.485 (3)
C3—C4	1.365 (3)	C26—C31	1.488 (3)
C3—H3	0.9300	C27—C28	1.358 (3)
C4—C5	1.385 (4)	C28—H28	0.9300
C4—H4	0.9300	C29—H129	0.9600
C5—C6	1.368 (3)	C29—H229	0.9600
C5—H5	0.9300	C29—H329	0.9600
C6—C7	1.387 (3)	C30—H130	0.9600
C6—H6	0.9300	C30—H230	0.9600
C8—C9	1.480 (3)	C30—H330	0.9600
C8—C13	1.490 (3)	C31—C32	1.382 (3)
C9—C10	1.364 (3)	C31—C36	1.390 (3)
C10—H10	0.9300	C32—C33	1.381 (4)
C11—H111	0.9600	C32—H32	0.9300
C11—H211	0.9600	C33—C34	1.364 (4)
C11—H311	0.9600	C33—H33	0.9300
C12—H112	0.9600	C34—C35	1.366 (4)
C12—H212	0.9600	C34—H34	0.9300
C12—H312	0.9600	C35—C36	1.368 (4)
C13—C14	1.381 (3)	C35—H35	0.9300
C13—C18	1.384 (3)	C36—H36	0.9300
			
C1—N1—C2	125.81 (18)	C15—C16—H16	120.2
C1—N1—HN1	117.1	C16—C17—C18	120.4 (3)
C2—N1—HN1	117.1	C16—C17—H17	119.8
C8—N2—C7	121.94 (18)	C18—C17—H17	119.8
C10—N3—C11	124.4 (2)	C13—C18—C17	121.0 (2)
C10—N3—C12	119.3 (2)	C13—C18—H18	119.5
C11—N3—C12	116.0 (2)	C17—C18—H18	119.5
C19—N4—C20	124.02 (18)	O2—C19—N4	120.08 (19)
C19—N4—HN4	118.0	O2—C19—C27	123.1 (2)
C20—N4—HN4	118.0	N4—C19—C27	116.86 (18)
C26—N5—C25	120.83 (18)	C21—C20—C25	119.4 (2)
C28—N6—C29	123.3 (2)	C21—C20—N4	118.8 (2)
C28—N6—C30	120.1 (2)	C25—C20—N4	121.8 (2)
C29—N6—C30	116.09 (19)	C22—C21—C20	121.2 (2)
O1—C1—N1	119.60 (19)	C22—C21—H21	119.4
O1—C1—C9	123.0 (2)	C20—C21—H21	119.4
N1—C1—C9	117.35 (18)	C21—C22—C23	119.8 (3)
C3—C2—C7	119.6 (2)	C21—C22—H22	120.1
C3—C2—N1	117.26 (19)	C23—C22—H22	120.1
C7—C2—N1	123.1 (2)	C24—C23—C22	119.6 (3)
C4—C3—C2	121.0 (2)	C24—C23—H23	120.2
C4—C3—H3	119.5	C22—C23—H23	120.2
C2—C3—H3	119.5	C23—C24—C25	121.3 (2)
C3—C4—C5	119.7 (2)	C23—C24—H24	119.4
C3—C4—H4	120.2	C25—C24—H24	119.4
C5—C4—H4	120.2	C24—C25—C20	118.6 (2)
C6—C5—C4	119.9 (2)	C24—C25—N5	117.4 (2)
C6—C5—H5	120.0	C20—C25—N5	123.9 (2)
C4—C5—H5	120.0	N5—C26—C27	125.0 (2)
C5—C6—C7	121.5 (2)	N5—C26—C31	116.26 (19)
C5—C6—H6	119.2	C27—C26—C31	118.60 (19)
C7—C6—H6	119.2	C28—C27—C19	113.94 (18)
C6—C7—C2	118.2 (2)	C28—C27—C26	128.89 (19)
C6—C7—N2	117.88 (19)	C19—C27—C26	116.65 (18)
C2—C7—N2	123.59 (19)	N6—C28—C27	131.3 (2)
N2—C8—C9	125.8 (2)	N6—C28—H28	114.4
N2—C8—C13	116.74 (18)	C27—C28—H28	114.4
C9—C8—C13	117.21 (19)	N6—C29—H129	109.5
C10—C9—C1	114.81 (18)	N6—C29—H229	109.5
C10—C9—C8	128.1 (2)	H129—C29—H229	109.5
C1—C9—C8	116.25 (18)	N6—C29—H329	109.5
N3—C10—C9	131.4 (2)	H129—C29—H329	109.5
N3—C10—H10	114.3	H229—C29—H329	109.5
C9—C10—H10	114.3	N6—C30—H130	109.5
N3—C11—H111	109.5	N6—C30—H230	109.5
N3—C11—H211	109.5	H130—C30—H230	109.5
H111—C11—H211	109.5	N6—C30—H330	109.5
N3—C11—H311	109.5	H130—C30—H330	109.5
H111—C11—H311	109.5	H230—C30—H330	109.5
H211—C11—H311	109.5	C32—C31—C36	117.7 (2)
N3—C12—H112	109.5	C32—C31—C26	121.9 (2)
N3—C12—H212	109.5	C36—C31—C26	120.4 (2)
H112—C12—H212	109.5	C33—C32—C31	120.9 (2)
N3—C12—H312	109.5	C33—C32—H32	119.5
H112—C12—H312	109.5	C31—C32—H32	119.5
H212—C12—H312	109.5	C34—C33—C32	120.4 (3)
C14—C13—C18	117.6 (2)	C34—C33—H33	119.8
C14—C13—C8	121.2 (2)	C32—C33—H33	119.8
C18—C13—C8	121.1 (2)	C33—C34—C35	119.3 (3)
C15—C14—C13	120.5 (2)	C33—C34—H34	120.3
C15—C14—H14	119.7	C35—C34—H34	120.3
C13—C14—H14	119.7	C34—C35—C36	121.0 (3)
C14—C15—C16	120.8 (3)	C34—C35—H35	119.5
C14—C15—H15	119.6	C36—C35—H35	119.5
C16—C15—H15	119.6	C35—C36—C31	120.7 (2)
C17—C16—C15	119.6 (3)	C35—C36—H36	119.6
C17—C16—H16	120.2	C31—C36—H36	119.6
			
C2—N1—C1—O1	155.3 (2)	C20—N4—C19—O2	−152.1 (2)
C2—N1—C1—C9	−25.2 (3)	C20—N4—C19—C27	27.3 (3)
C1—N1—C2—C3	−133.3 (2)	C19—N4—C20—C21	128.2 (2)
C1—N1—C2—C7	49.3 (3)	C19—N4—C20—C25	−53.5 (3)
C7—C2—C3—C4	−2.5 (4)	C25—C20—C21—C22	3.5 (4)
N1—C2—C3—C4	179.9 (2)	N4—C20—C21—C22	−178.2 (2)
C2—C3—C4—C5	0.5 (4)	C20—C21—C22—C23	−1.8 (4)
C3—C4—C5—C6	2.1 (5)	C21—C22—C23—C24	−1.2 (4)
C4—C5—C6—C7	−2.7 (4)	C22—C23—C24—C25	2.6 (4)
C5—C6—C7—C2	0.6 (4)	C23—C24—C25—C20	−0.9 (4)
C5—C6—C7—N2	174.1 (2)	C23—C24—C25—N5	−176.5 (2)
C3—C2—C7—C6	2.0 (3)	C21—C20—C25—C24	−2.1 (3)
N1—C2—C7—C6	179.4 (2)	N4—C20—C25—C24	179.7 (2)
C3—C2—C7—N2	−171.1 (2)	C21—C20—C25—N5	173.2 (2)
N1—C2—C7—N2	6.3 (3)	N4—C20—C25—N5	−5.0 (3)
C8—N2—C7—C6	144.1 (2)	C26—N5—C25—C24	−138.0 (2)
C8—N2—C7—C2	−42.8 (3)	C26—N5—C25—C20	46.6 (3)
C7—N2—C8—C9	−0.2 (3)	C25—N5—C26—C27	−4.4 (3)
C7—N2—C8—C13	174.40 (18)	C25—N5—C26—C31	179.93 (19)
O1—C1—C9—C10	−33.0 (3)	O2—C19—C27—C28	35.1 (3)
N1—C1—C9—C10	147.5 (2)	N4—C19—C27—C28	−144.2 (2)
O1—C1—C9—C8	137.2 (2)	O2—C19—C27—C26	−137.4 (2)
N1—C1—C9—C8	−42.3 (3)	N4—C19—C27—C26	43.3 (3)
N2—C8—C9—C10	−130.9 (2)	N5—C26—C27—C28	129.4 (3)
C13—C8—C9—C10	54.6 (3)	C31—C26—C27—C28	−55.0 (3)
N2—C8—C9—C1	60.4 (3)	N5—C26—C27—C19	−59.4 (3)
C13—C8—C9—C1	−114.2 (2)	C31—C26—C27—C19	116.1 (2)
C11—N3—C10—C9	5.8 (4)	C29—N6—C28—C27	−5.0 (4)
C12—N3—C10—C9	179.3 (3)	C30—N6—C28—C27	−176.9 (2)
C1—C9—C10—N3	−174.9 (2)	C19—C27—C28—N6	173.6 (2)
C8—C9—C10—N3	16.2 (4)	C26—C27—C28—N6	−15.0 (4)
N2—C8—C13—C14	21.1 (3)	N5—C26—C31—C32	165.7 (2)
C9—C8—C13—C14	−163.9 (2)	C27—C26—C31—C32	−10.3 (3)
N2—C8—C13—C18	−155.1 (2)	N5—C26—C31—C36	−15.5 (3)
C9—C8—C13—C18	19.9 (3)	C27—C26—C31—C36	168.6 (2)
C18—C13—C14—C15	0.1 (4)	C36—C31—C32—C33	1.0 (4)
C8—C13—C14—C15	−176.3 (2)	C26—C31—C32—C33	179.9 (3)
C13—C14—C15—C16	−0.8 (4)	C31—C32—C33—C34	−1.2 (5)
C14—C15—C16—C17	1.0 (4)	C32—C33—C34—C35	0.6 (6)
C15—C16—C17—C18	−0.5 (4)	C33—C34—C35—C36	0.3 (6)
C14—C13—C18—C17	0.4 (4)	C34—C35—C36—C31	−0.5 (5)
C8—C13—C18—C17	176.8 (2)	C32—C31—C36—C35	−0.1 (4)
C16—C17—C18—C13	−0.2 (4)	C26—C31—C36—C35	−179.0 (3)

### Hydrogen-bond geometry (Å, º)

**Table:**

*D*—H···*A*	*D*—H	H···*A*	*D*···*A*	*D*—H···*A*
N1—H*N*1···O2^i^	0.86	2.17	2.861 (2)	137
N4—H*N*4···O1^ii^	0.86	2.41	2.939 (2)	121

Symmetry codes: (i) *x*+1, *y*+1, *z*; (ii) *x*−1, *y*−1, *z*.
